# Human-Derived Physiological Heat Shock Protein 27 Complex Protects Brain after Focal Cerebral Ischemia in Mice

**DOI:** 10.1371/journal.pone.0066001

**Published:** 2013-06-13

**Authors:** Shinichiro Teramoto, Hideki Shimura, Ryota Tanaka, Yoshiaki Shimada, Nobukazu Miyamoto, Hajime Arai, Takao Urabe, Nobutaka Hattori

**Affiliations:** 1 Department of Neurology, Juntendo University School of Medicine, Tokyo, Japan; 2 Department of Neurosurgery, Juntendo University School of Medicine, Tokyo, Japan; 3 Department of Neurology at Juntendo University Urayasu Hospital, Juntendo University School of Medicine, Chiba, Japan; 4 Institute for Environment and Gender-Specific Medicine, Juntendo University Graduate School of Medicine, Chiba, Japan; University of Queensland, Australia

## Abstract

Although challenging, neuroprotective therapies for ischemic stroke remain an interesting strategy for countering ischemic injury and suppressing brain tissue damage. Among potential neuroprotective molecules, heat shock protein 27 (HSP27) is a strong cell death suppressor. To assess the neuroprotective effects of HSP27 in a mouse model of transient middle cerebral artery occlusion, we purified a “physiological” HSP27 (hHSP27) from normal human lymphocytes. hHSP27 differed from recombinant HSP27 in that it formed dimeric, tetrameric, and multimeric complexes, was phosphorylated, and contained small amounts of αβ-crystallin and HSP20. Mice received intravenous injections of hHSP27 following focal cerebral ischemia. Infarct volume, neurological deficit scores, physiological parameters, and immunohistochemical analyses were evaluated 24 h after reperfusion. Intravenous injections of hHSP27 1 h after reperfusion significantly reduced infarct size and improved neurological deficits. Injected hHSP27 was localized in neurons on the ischemic side of the brain. hHSP27 suppressed neuronal cell death resulting from cytochrome c-mediated caspase activation, oxidative stress, and inflammatory responses. Recombinant HSP27 (rHSP27), which was artificially expressed and purified from *Escherichia coli*, and dephosphorylated hHSP27 did not have brain protective effects, suggesting that the phosphorylation of hHSP27 may be important for neuroprotection after ischemic insults. The present study suggests that hHSP27 with posttranslational modifications provided neuroprotection against ischemia/reperfusion injury and that the protection was mediated through the inhibition of apoptosis, oxidative stress, and inflammation. Intravenously injected human HSP27 should be explored for the treatment of acute ischemic strokes.

## Introduction

Ischemic brain injury is a major health problem. Despite numerous clinical trials, many neuroprotective therapies have failed [1]. Protecting brain tissue from ischemic injury is a considerable challenge in stroke treatment strategies. However, not all brain cells die immediately after an ischemic event. Surrounding the core of severely and rapidly injured brain tissue, cell death spreads slowly in a heterogeneous region called the penumbra, which is salvageable [2]. While numerous preclinical studies demonstrated that neuroprotective strategies significantly reduce the ischemic penumbra [3], many strategies have failed in clinical trials for several reasons [4]. For example, reactions to compounds and peptides may differ between test animals and humans. We hypothesized that endogenous human proteins should not evoke adverse reactions and might be ideal neuroprotective molecules for treating ischemic stroke patients.

Neuronal injury after cerebral ischemia involves a complex series of cellular stresses, including oxidative stress, inflammation, and apoptosis, all of which can lead to cell death [5], [6]. Thus, multifunctional molecules that suppress cell death, are anti-apoptotic, and scavenge free radicals are ideal neuroprotective agents. Heat shock protein 27 (HSP27) provides robust cellular protection, is an adenosine triphosphate-independent chaperone, a free radical scavenger, and is anti-apoptotic [7]. HSP27 undergoes various posttranslational modifications, including phosphorylation and oligomerization, and interacts with other small heat shock proteins, such as αβ-crystallin and HSP20 [8], influencing its oligomeric state and regulating its function [9], [10].

HSP27-transgenic cell and mouse lines exhibit numerous cytoprotective effects in *in vivo* models of various diseases, including cardiac ischemia [11], [12], kainate-induced hippocampal cell death [13], and nerve injury [14], in the tau model of Alzheimer disease [15], [16], and in the SOD1G93A model of amyotrophic lateral sclerosis [17]. HSP27-transgenic mice exhibit reduced infarcts after transient cerebral ischemia [18], and viral delivery of HSP27 and intraperitoneal injection of PEP1-HSP27, but not HSP27 recombinant protein, into ischemic animal models are also protective [19], [20]. Finally, endogenous induction of HSP27 was observed in ischemia-surviving cells [21] and in ischemic preconditioning models [22], [23], suggesting that HSP27 is associated with cellular survival following cerebral ischemia. Phosphorylation and oligomerization of HSP27 are both essential for mediating neuroprotection against ischemic neuronal injury in HSP27 transgenic mouse models [24]. All of which suggest that HSP27 is a strong candidate molecule for brain protection against ischemic insults, and led us to hypothesize that posttranslationally modified HSP27 might be a better treatment therapy than non-modified recombinant HSP27. We tested this hypothesis by purifying HSP27 from human lymphocytes (hHSP27) and demonstrated that it attenuated ischemic brain damage in a mouse model of transient middle cerebral artery occlusion (MCAO).

## Materials and Methods

### HSP27 Antibodies

We generated 2 anti-HSP27 rabbit polyclonal antibodies: anti-HSP27-N1 against the 15-mer sequence MTERRVPFSLLRGPC at the N-terminal domain of human HSP27 and anti-HSP27-C1 against the 15-mer sequence CGGPEAAKSDETAAK at the C-terminal domain of human HSP27.

### Human Physiological HSP27 Preparation

Heparinized human peripheral blood (40 mL) was obtained from two normal control subjects and separated by density gradient centrifugation in Lympholyte-H (Cedarlane Laboratories Ltd., Hornby, Ontario, Canada) according to the manufacturer’s instructions. Cells were lysed in lysis buffer (50 mmol/L Tris-HCl, pH 7.5, 150 mmol/L NaCl, 5 mmol/L EDTA, 0.5% sodium deoxycholate, and 0.1 mmol/L phenylmethylsulfonyl fluoride) with a Dounce homogenizer, and the lysate was centrifuged at 10,000×*g* for 1 h. The supernatant was applied to an HSP27-N1 antibody affinity column, and the column was washed with lysis buffer. HSP27 was eluted by peptide antigen (10 mg/mL) for the HSP27-N1 antibody. The eluate was further applied to an HSP27-C1 antibody affinity column, and the column was washed with lysis buffer. HSP27 was eluted by a 10-mg/mL excess amount of HSP27-C1 antibody peptide antigen. HSP27 was separated from the peptide with Amicon Ultra-10 centrifugal filter units (Millipore, Billerica, MA, USA). The purity of the hHSP27 protein was over 95%. The investigation conforms to the principles outlined in the Declaration of Helsinki, and was reviewed and approved by the Juntendo University Ethics Committee. Written informed consent was obtained from all participants.

### MS/MS Identification of hHSP27

hHSP27 was separated by native or SDS-polyacrylamide gel electrophoresis (PAGE). Proteins were stained with Coomassie Brilliant Blue. For protein spot analysis, including MS (Mass Spectrometry)/MS and MASCOT search analysis, we used the technical services of ProPhoenix Co., Ltd. (Hiroshima, Japan).

### Experimental Protocol

Animal procedures were approved by the Animal Care Committee of Juntendo University. Eight-week-old adult male C57BL/6 mice weighing 20–23 g were housed under controlled lighting and provided food and water *ad libitum*. Mice were subjected to transient, 1-h MCAO, and then randomly divided into 3 groups: (1) an hHSP27 group that received tail-vein injections of hHSP27 after reperfusion, (2) a control group that received intravenous injections of 50 µg of bovine serum albumin (BSA), and (3) a sham-operated group that underwent the same procedure without MCAO. During this procedure, body temperature was maintained at 37.0±0.5°C with a heating pad. Systolic blood pressure was monitored by a noninvasive tail-cuff system (Softron BP-98A NIBP, Softron Co., Ltd.) in conscious mice. The selected dose and schedule of hHSP27 treatments were based on preliminary experiments that used 5 or 50 µg/mouse hHSP27 administered 0 (immediately), 1, 3, or 6 h after reperfusion (n = 3 in each group). Regional cerebral blood flow was measured by laser Doppler flowmetry before, during, and after MCAO, and before the mice were sacrificed. 24 h after reperfusion, mice were anesthetized by intraperitoneal injections of 50 mg/kg pentobarbital and decapitated. To evaluate infarct area and volume, brain slices were stained with cresyl violet or 2,3,5-triphenyltetrazolium chloride, scanned with AxioVision software (Carl Zeiss MicroImaging GmbH), and measured using the ImageJ program (NIH, http://rsb.info.nih.gov/nih-image/) [25].

### Neurological Evaluation

Neurological functions were evaluated by the following modified scoring system: 0, no observable neurological deficits (normal); 1, failure to extend forepaw when entire body is lifted by tail (mild); 2, circling to contralateral side (moderate); and 3, loss of walking or righting reflex (severe) [26]. Three mice were tested in each group, and each mouse was subjected to 3 rounds of each test. The observers of the behavioral tests were blinded to the treatment groups, and mice of the various groups were randomized during a given testing period.

### hHSP27 and HSP27 Antibody, HSP27 Elution Peptide Administration or Recombinant HSP27 Administration

Mice received intravenous injections of 50 µg of hHSP27 mixed with 50 or 500 µg of HSP27-N1 or -C1 antibody 1 h after reperfusion (n = 3 in each group), 5 or 50 µg of HSP27-N1 and -C1 peptides, which were used in the elution, intravenously 1 h after reperfusion (n = 3 in each group), or 50 µg of recombinant HSP27 (rHSP27; *Acris Antibodies GmbH*) 1 h after reperfusion (n = 3).

### Dephosphorylated hHSP27 Administration

hHSP27 was dephosphorylated by active recombinant protein phosphatase 2A (Millipore) [16]. Mice received intravenous injections of 50 µg of dephosphorylated hHSP27 1 h after reperfusion (n = 3).

### Identification of Transition in Brain Parenchyma by Intravenous hHSP27 Injection

hHSP27 was conjugated with fluorescein isothiocyanate (FITC) according to the manufacturer’s protocol (KPL, Inc.). Mice were administered 50 µg of FITC-hHSP27 intravenously 1 h after reperfusion and anesthetized with pentobarbital 30 min after the injection. Their brains were immediately removed, soaked in Tissue-Tek® OCT™ Compound (SAKURA, Netherland), and frozen on liquid nitrogen. Coronal sections (20 µm) were cut on a cryostat (CM 1900, Leica Biosystems Nussloch GmbH, Nussloch, Germany). The sections were immediately, or after incubation with Alexa Fluor® 555 Conjugated anti-NeuN antibody (Millipore), mounted with Vectashield mounting medium (Vector Laboratories, Inc., Burlingame, CA, USA). The sections were examined with an LSM 510 confocal laser scanning microscope (Carl Zeiss MicroImaging GmbH).

### TUNEL Assay

For *in situ* DNA fragmentation detection, terminal deoxynucleotidyl transferase-mediated dUTP-biotin nick-end labeling (TUNEL) was carried out with an *in situ* cell death detection kit (TMR Red, Roche Diagnostics GmbH) [27].

### Fractionation of Mouse Brain

Twenty-four hours after reperfusion, a brain sample was harvested from ischemic regions of the cortex and striatum on the operated side of each mouse and placed in ice-cold synaptosome homogenizing buffer (320 mmol/L sucrose, 4 mmol/L HEPES, pH 7.4) with Complete Mini, EDTA-free, and phosphatase inhibitor cocktails I and II (Sigma-Aldrich Co.). Tissues were homogenized with a glass-Teflon homogenizer (12 up/down strokes, 900 rpm). The homogenized sample was centrifuged at 3,000×*g* for 5 min (step 1), and the supernatant was centrifuged at 12,000×*g* for 10 min (step 2). The resulting pellet was resuspended in isolation media and centrifuged at 3,000×*g* for 5 min to remove nuclear contamination (step 3). The supernatant from step 3 was centrifuged at 12,000×*g* for 10 min (step 4). Steps 3 and 4 were repeated twice to further purify the mitochondrial fraction. The resulting pellet from the 12,000×*g* spin was the mitochondria-enriched fraction. The supernatant obtained from step 2 was centrifuged at 70,000×*g* for 60 min (step 5). The resulting supernatant was the soluble cytosolic fraction. The pellet fractions were resuspended in isolation media. The purity of the fractions was tested by immunoblotting with a rabbit Tom20 antibody (mitochondrial marker; 1∶5,000; Santa Cruz Biotechnology, Inc.). Protein loading was confirmed in cytosolic fractions by immunoblotting with mouse anti-actin antibody (1∶10,000; Millipore). The protein concentration in each fraction was determined with a Pierce BCA protein assay kit (Thermo Fisher Scientific, Inc., Rockford, IL, USA), and the fractions were subjected to immunoblotting with anti-cytochrome c antibody (1∶1,000).

### Immunohistochemistry

Immunohistochemistry was performed on 20-µm free-floating sections. Sections were stained overnight using HSP27 C-1, rabbit anti-cytochrome c (1∶100; Cell Signaling Technology, Inc., Beverly, MA, USA), rabbit anti-cleaved caspase-9 (1∶50; Cell Signaling Technology, Inc.), rabbit anti-cleaved caspase-3 (1∶200; Cell Signaling Technology, Inc.), mouse anti-8-hydroxydeoxyguanosine (8-OHdG; 1∶100; Japan Institute for the Control of Aging, Shizuoka, Japan), mouse anti-4-hydroxy-2-hexenal (HHE; 1∶100; Japan Institute for the Control of Aging), rabbit anti-ionized calcium-binding adapter molecule-1 (Iba-1; 1∶500; Wako Pure Chemical Industries, Ltd., Osaka, Japan), and rabbit anti-glial fibrillary acidic protein (GFAP; 1∶500; Dako North America, Inc., Carpinteria, CA, USA) antibodies. Sections were then incubated with biotinylated secondary antibodies (1∶300; Vector Laboratories, Inc.) and subsequently processed with avidin-biotinylated peroxidase complex (Vectastain ABC kit; 1∶400; Vector Laboratories, Inc.).

### PAGE and Immunoblotting

Each mouse brain sample was obtained from the ischemic region of cortex and striatum on the operated side 24 h after reperfusion. Frozen human brain (78-year-old who died of bladder cancer) was obtained from the temporal cortex. SDS-PAGE experiments were performed with the NuPAGE Novex Bis-Tris Gel system according to the manufacturer’s instructions (Invitrogen, Carlsbad, CA). The most frequently used SDS gel was a 4–12% gradient gel. Native–PAGE experiments were performed with the NativePAGE Novex Bis-Tris Gel System according to the manufacturer’s instructions. The most frequently used native gel was a 4–16% gradient gel. To this solution, an additional detergent to be tested was added at a final concentration of 0.4% [1.0% in the case of *n*-octyl-β-d-glucoside (β-OG)] and incubated for 10 min prior to blue native–PAGE. To each lane of a native gel, 3–5 µg of protein were loaded. Anode buffer was made by diluting the 20×NativePAGE running buffer (Invitrogen, Carlsbad, CA), and the cathode buffer by mixing the NativePAGE running buffer with Cathode Buffer additive (Coomassie Blue G-250 dye, Invitrogen) according to the manufacturer’s instructions. For BN–PAGE with membrane proteins, the concentration of the blue dye was 0.02%(*w*/*v*), which is tenfold higher than that for soluble proteins. The gel was stained using the Colloidal Blue Staining Kit (Invitrogen). Block Ace (Daiichi Kogyo Seiyaku, Co., Ltd., Gifu, Japan) or PBS containing 0.05% Tween-20 (Sigma-Aldrich Co.) was used for blocking. Membranes with transferred proteins were incubated overnight with antibodies against HHE (1∶1,000; Cosmo Bio Co., Ltd Carlsbad CA), and reacted with horseradish peroxidase-conjugated secondary antibody (1∶5,000; GE Healthcare Life Sciences, Buckinghamshire, UK). Immunoreactive bands were visualized with an enhanced chemiluminescence kit (GE Healthcare Life Sciences). Protein loading of lanes was assessed with α-tubulin immunoreactivity (53 kDa).

### Cell Counts

In the immunohistochemical analyses, positively stained cells in the ischemic boundary zone (IBZ) adjacent to the ischemic core (0.25 mm^2^) were counted in 5 sections from each of 3 mice were counted using AxioVision by an investigator who was blinded to the experimental groups. All values are expressed as mean ± SEM.

### Statistical Analysis

One-way analysis of variance and Fisher’s exact test protected least significant difference tests were used to determine the significance of differences between the groups. *P* values less than 0.05 indicated statistical significance.

## Results

### Purification of Human HSP27

**Figure 1:**
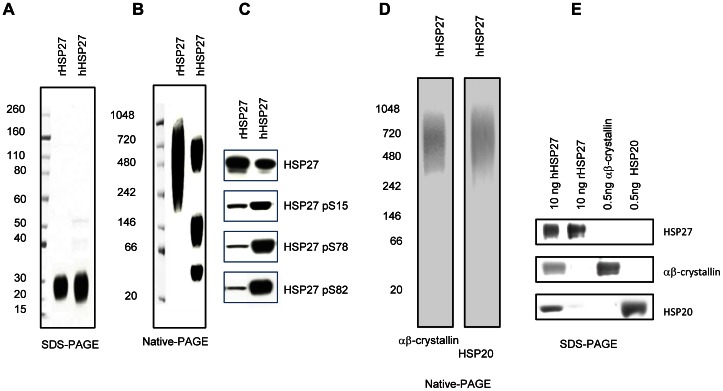
Characterization of hHSP27. ***A–B,*** Isolated human heat shock protein 27 (hHSP27) or recombinant HSP27 (rHSP27) proteins (1 µg or 10 µg) were separated by SDS-PAGE (***A***) and native-PAGE (***B***) and stained with Coomassie brilliant blue. ***C,*** hHSP27 proteins were immunoblotted with antibodies against HSP27, phosphorylated S15 HSP27, S78 HSP27, and S82 HSP27. ***D,*** hHSP27 proteins were separated by native-PAGE and immunoblotted with antibodies against αβ-crystallin and HSP20. ***E,*** rHSP27 (10 ng), hHSP27 (10 ng), αβ-crystallin (5 ng), and HSP20 (5 ng) were separated by SDS-PAGE followed by immunoblotting with antibodies against HSP27, αβ-crystallin, and HSP20.

To examine the effects of HSP27 on ischemic injury, we initially injected recombinant HSP27 (rHSP27) into ischemic model mice, but did not observe any infarct suppression (data not shown). Because rHSP27 is not phosphorylated and occurs in large oligomers, we decided to use human physiological HSP27 for subsequent studies because it is phosphorylated (at S15, S78, and S82) and reorganizes from large oligomers into dimers, tetramers, and multimers in response to many insults and interacts with other small HSPs [7]. To obtain physiological HSP27, we purified HSP27 from human lymphocytes (hHSP27) with 2 human-specific HSP27-antibody affinity columns. The resulting protein purity was more than 95% (Figure 1A). We found more dimeric, tetrameric, and multimeric configurations and fewer large oligomers of hHSP27 than those of rHSP27 (Figure 1B), and increased phosphorylation (Figure 1C). A mass spectrometric analysis revealed that a high molecular weight hHSP27 multimer contained HSP27 and αβ-crystallin and HSP20. We also identified the peptides, RVPFSLLRGPSWDPFRDWYPHSRLFDQAFGLPR, VSLDVNHFAPDELTVK, KYTLPPGVDPTQVSSSLSPEGTLTVEAPMPK, LATQSNEITIPVTFESR, and AQLGGPEAAKSDETAAK, which are consistent with the HSP27 protein sequence (gi662841); MDIAIHHPWIR, RPFFPFHSPSR, APSWFDTGLSEMR and IPADVDPLTITSSLSSDGVLTVNGPR, which are consistent with the αβ-crystallin protein sequence (gi2845682); and MEIPVPVQPSWLR and HEERPDEHGFVAR, which are consistent with the HSP20 protein sequence (gi2477511). The hHSP27 dimer and tetramer contained only HSP27 without αβ-crystallin and HSP20. Immunoblot analysis revealed that the high molecular weight hHSP27 multimer contained αβ-crystallin and HSP20 (Figure 1D). Both immunoblot and mass spectrometric analyses (data not shown) revealed that rHSP27 contained only HSP27 and not αβ-crystallin and HSP20. Ten nanograms of hHSP27 contained less than 0.5 ng each of αβ-crystallin and HSP20, that is, the amount of HSP27 contained in the hHSP27 was more than 20 times that of αβ-crystallin and HSP20 (Figure 1E). The amounts of αβ-crystallin and HSP20 were determined by comparing them with known amounts of their respective commercial recombinant proteins. We also chose to use hHSP27 in subsequent studies, because hHSP27 subjected to various physiological posttranslational modifications may influence function.

### hHSP27 Attenuates Ischemic Brain Damage

**Figure 2:**
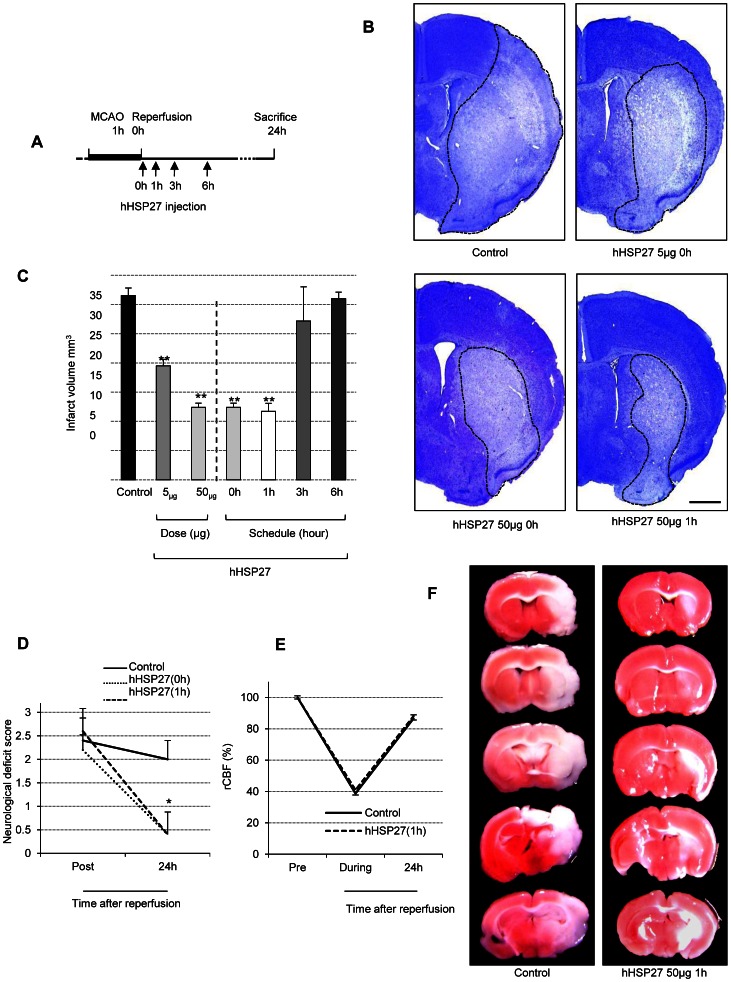
hHSP27 attenuates ischemic brain damage. ***A,*** Schematic of hHSP27 injection schedules for determining the HSP27 treatment protocol. ***B,*** Photomicrographs of cresyl violet-stained infarcts in controls, the 5-µg hHSP27 group (0 h), 50-µg hHSP27 group (0 h), and 50-µg hHSP27 group (1 h), 24 h after reperfusion. Infarct areas are circumscribed with dotted lines. Scale bar = 1 mm. ***C,*** Infarct volumes at various doses (given at 0 h) and schedules (50 µg doses) of hHSP27. ***D,*** Neurological deficit scores in controls, the 50-µg hHSP27 group (0 h), and the 50-µg hHSP27 group (1 h) immediately (Post) and 24 h following reperfusion. ***E,*** Temporal changes in regional cerebral blood flow (rCBF), before (Pre), during, and 24 h after MCAO. ***F,*** Photomicrographs of infarct areas stained with 2,3,5-triphenyltetrazolium chloride in controls and the 50-µg hHSP27 group (1 h) 24 h after reperfusion. Data are presented as mean±SEM of 3 mice (***B,E***) and 5 mice (***D***) in each group. *P<0.05, **P<0.001 vs. controls. hHSP27, human heat shock protein; MCAO, middle cerebral artery occlusion.

**Figure 3:**
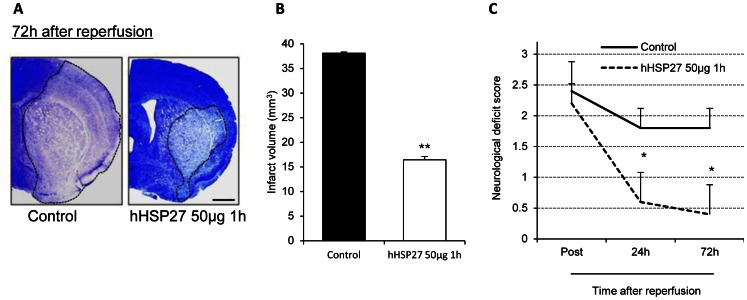
Neuroprotective effects of hHSP27 against ischemic/reperfusion injury 72 h after reperfusion. ***A,*** Photomicrographs of infarct areas stained with cresyl violet in control and hHSP27 groups 72 h after reperfusion. Infarct areas are circumscribed with dotted lines. Scale bar = 1 mm. ***B,*** Infarct volumes in control and hHSP27 groups. ***C,*** Neurological deficit scores in control and hHSP27 groups. Data are presented as mean±SEM of 3 mice (***B***) and 5 mice (***C***) in each group. *P<0.05, **P<0.001 vs. controls.

The HSP27 treatment protocol was first determined in preliminary experiments. Ischemic mice (see Methods) were intravenously injected with either hHSP27 (5 or 50 µg) or BSA (50 µg) 0, 1, 3, or 6 h after reperfusion (Figure 2A), and infarct volumes were measured in cresyl violet-stained sections made 24 h after reperfusion (Figure 2B). Infarct volume was reduced by 37% in mice treated 0 h after reperfusion with 5 µg of hHSP27 (19.49±1.12 mm^3^, *P*<0.001, n = 5) and by 61% in those treated with 50 µg of hHSP27 (12.39±0.73 mm^3^, *P*<0.001, n = 5) vs. BSA-treated controls (31.55±1.28 mm^3^; n = 5, Figure 2B,C). Infarct volume tended to be reduced more when the 50-µg dose was administered 1 h after reperfusion (63% reduction; 11.71±1.36 mm^3^, *P*<0.001, n = 5); there was only a slight reduction at 3 h and no difference at 6 h after reperfusion vs. controls (Figure 2C). The hHSP27 group showed better functional recoveries [hHSP27 (0 h): *P* = 0.004, hHSP27 (1 h): *P* = 0.004] than controls (Figure 2D). There was no difference in regional cerebral blood flow between the treated and control groups (Figure 2E). Based on these findings, in the remaining experiments, we injected 50 µg of hHSP27 1 h after reperfusion because it was most effective in reducing infarct volume (Figure 2F). Significant reductions in infarct volume and neurological deficits were also found 72 h after reperfusion in mice injected with 50 µg of hHSP27 at 1 h (16.43±0.69 mm^3^ *P*<0.001, n = 3) vs. controls (38.09±0.24 mm^3^ n = 3) (Figure 3A,B,C).

**Figure 4:**
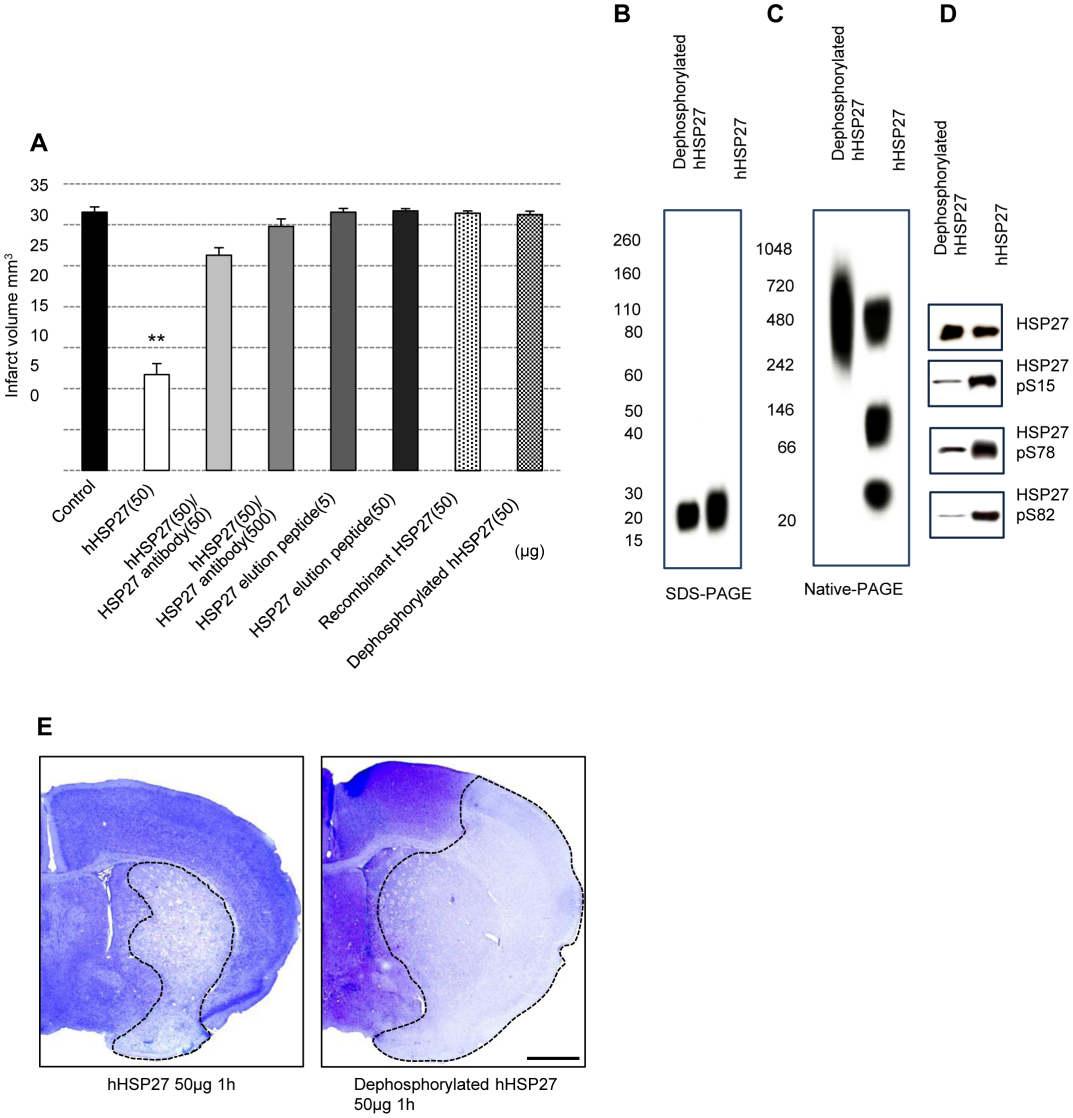
Anti-HSP27 antibody and dephosphorylation inhibit hHSP27 neuroprotective effects. ***A,*** Infarct volumes in control, hHSP27 (50 µg), hHSP27 plus HSP27 antibody cocktails, HSP27 elution peptide, recombinant HSP27, and dephosphorylated hHSP27 groups. Data are means±SEM of 3 mice in each group. **P<0.001 vs. controls. ***B– D,*** Dephosphorylated and phosphorylated hHSP27 proteins were separated by SDS-PAGE (***B***) and native-PAGE (***C***), stained with Coomassie brilliant blue (***B,C***), and immunoblotted with anti-phosphorylated S15 HSP27, S78 HSP27, and S82 HSP27 antibodies (***D***). ***E***, Photomicrographs of infarct areas stained with cresyl violet in hHSP27 and dephosphorylated hHSP27 groups prepared 24 h after reperfusion. Scale bar = 1 mm. hHSP27, human heat shock protein.

To exclude the possibility that molecules co-purified with HSP27, such as αβ-crystallin and HSP20, attenuated ischemic brain damage, we administered hHSP27 in the presence of HSP27-N1 and -C1 antibodies or HSP27-elution peptides (HSP27-N1 and -C1 peptides), instead of HSP27. The co-administration of hHSP27 and HSP27 antibody (50 µg hHSP27 and 50 µg HSP27 antibodies: 26.28±0.93 mm^3^ n = 3; 50 µg hHSP27 and 500 µg HSP27 antibodies: 29.79±0.93 mm^3^ n = 3) resulted in infarct volumes that were not significantly different from control (Figure 3A). The HSP27 antibody inhibited the protective effects of hHSP27, indicating hHSP27 as the source of cellular protection and not other co-purified molecules. The administration of HSP27 elution peptides had no effect on ischemic brain damage (Figure 4A).

Nonphosphorylated rHSP27 purified from *Escherichia coli* also did not reduce infarct volume (50 µg rHSP27∶31.41±0.27 mm^3^; Figure 4A). Treating hHSP27 with protein phosphatase 2A (PP2A) [16], which dephosphorylates hHSP27 at S15, S78, and S82, increased the large oligomers and decreased the dimers, tetramers, and multimers (Figure 4B, C, D). Dephosphorylated hHSP27 did not reduce infarct volume (50 µg dephosphorylated hHSP27∶31.24±0.41 mm^3^; Figure 4A and E). These results indicated that physiologically phosphorylated hHSP27 elicited neuroprotection.

### Localization of (FITC)-hHSP27 in Brain Parenchyma after Injection

**Figure 5:**
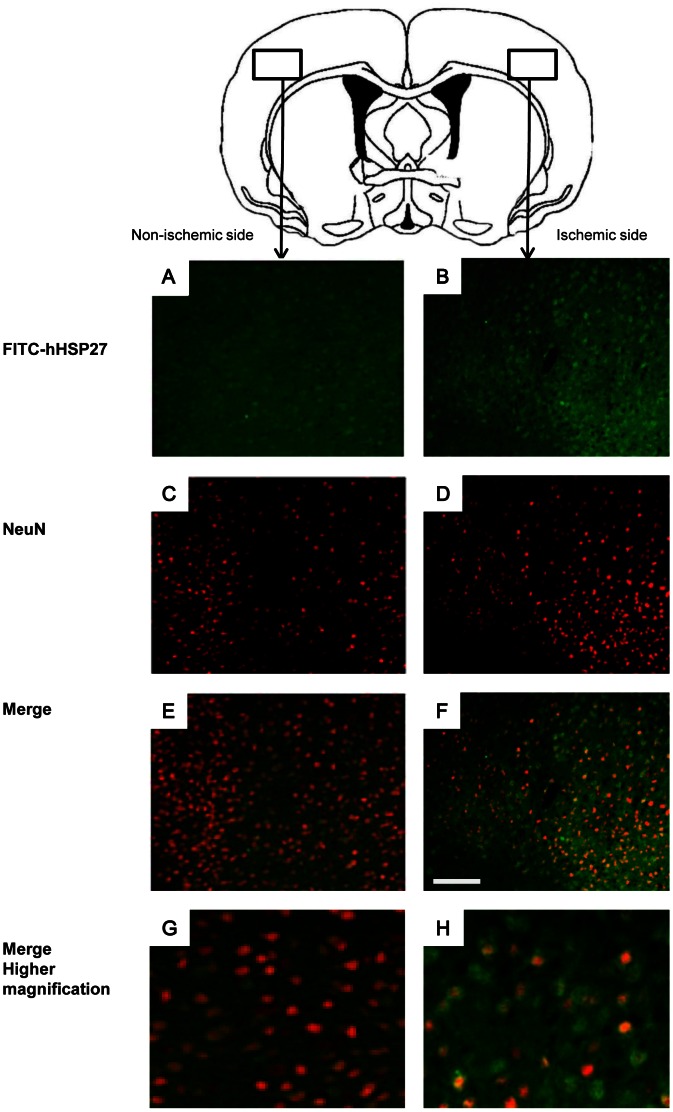
Localization of injected FITC-hHSP27 on the ischemic and non-ischemic sides of mouse brain. *****A–H,***** FITC-hHSP27 (*green*, ***A,B***); NeuN, a neuronal marker protein (*red*, ***C,D***); merge (***E–H***), FITC, fluorescein isothiocyanate. Scale bar = 100 um.

To obtain direct evidence of hHSP27 localization in brain, we injected fluorescein isothiocyanate (FITC)-hHSP27. To preserve the FITC-hHSP27 signal, we made fresh frozen sections. FITC-hHSP27 was diffusely localized in brain (Fig. 5A,B). FITC-HSP27 also localized in neuronal marker protein NeuN-positive cells (Figure 5C–H), indicating that FITC-hHSP27 was localized in neurons. FITC-hHSP27 was also localized in NeuN-negative cells. We found larger amounts of FITC-HSP27 on the ischemic side than on the non-ischemic side of brain.

### hHSP27 Suppressed Apoptotic Cell Death, Oxidative DNA Damage, Lipid Peroxidation and Glial Activation

**Figure 6:**
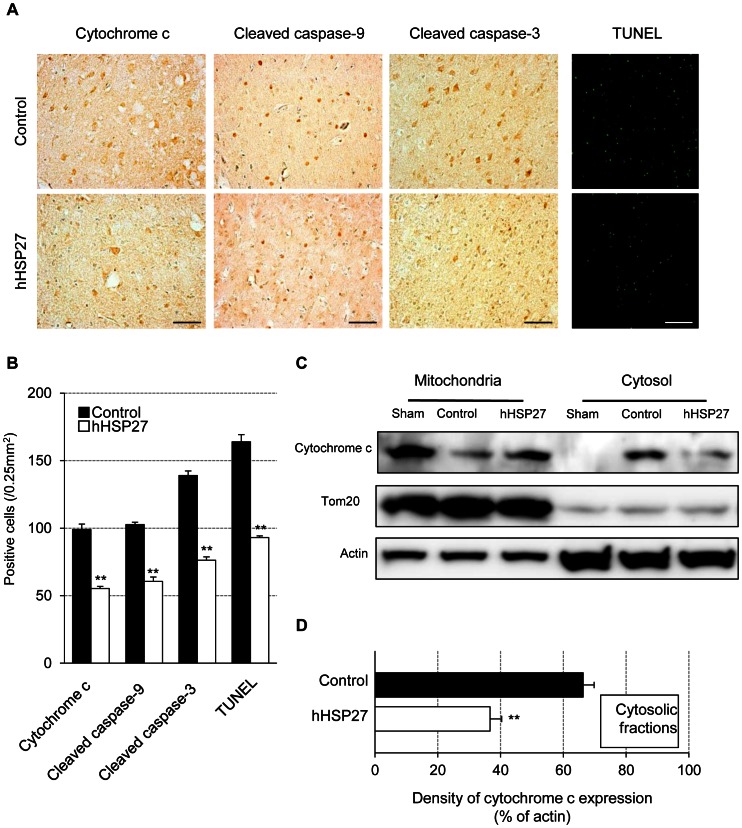
Effects of hHSP27 on cell death. ***A,*** Photomicrographs of anti-cytochrome c, anti-cleaved caspase-9, anti-cleaved caspase-3, and TUNEL staining in the infarct boundary zones in controls and the hHSP27-treated group prepared 24 h after reperfusion. Scale bars = 50 µm. ***B,*** Number of cytochrome c-, cleaved caspase-9-, cleaved caspase-3-, and TUNEL-positive cells. ***C,*** Immunoblots of cytochrome c, Tom20 (mitochondrial marker), and actin, 24 h after reperfusion. ***D,*** Densitometric analysis of cytochrome c protein in cytosolic fractions of isolated hHSP27. Data are means±SEM (***B,D***). **P<0.001 vs. controls. TUNEL, terminal deoxynucleotidyl transferase-mediated dUTP-biotin nick-end labeling; hHSP27, human heat shock protein.

**Figure 7:**
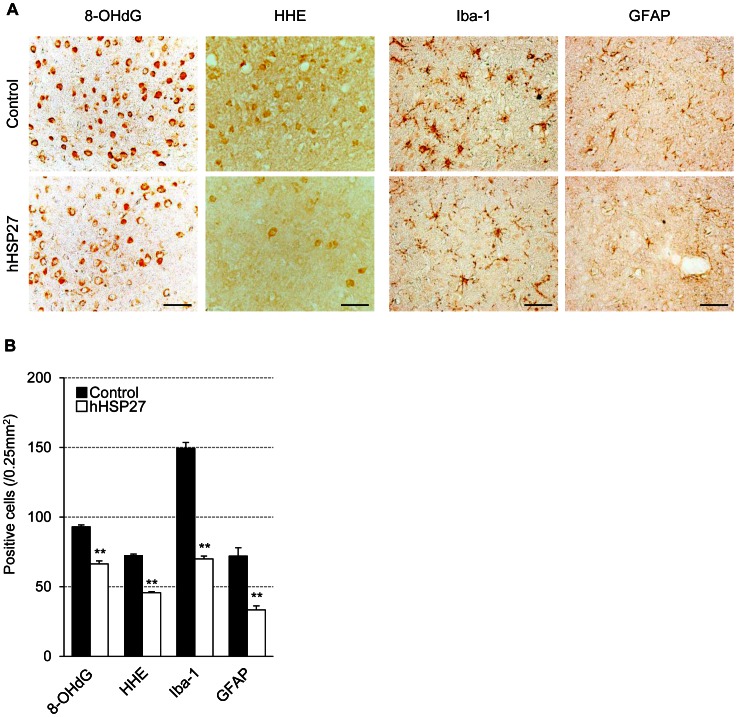
Effects of hHSP27 on oxidative stress and inflammatory response. ***A,*** Photomicrographs of 8-OHdG-, HHE-, Iba-1-, and GFAP-immunostaining in the infarct boundary zones in the control and hHSP27 groups 24 h after reperfusion. Bars = 50 µm. ***B,*** Numbers of 8-OHdG-, HHE–, Iba-1-, and GFAP-positive cells in control and hHSP27-treated mice. Data are means±SEM (***B***). **P<0.001 vs. controls. 8-OHdG, 8-hydroxydeoxyguanosine; HHE, 4-hydroxy-2-hexenal; Iba-1, ionized calcium-binding adapter molecule-1; GFAP, glial fibrillary acidic protein; hHSP27, human heat shock protein.

The numbers of cells immunopositive for cytochrome c, cleaved caspase-9, and cleaved caspase-3, and the number of terminal deoxynucleotidyl transferase-mediated dUTP-biotin nick-end labeling (TUNEL)-positive cells 24 h after reperfusion were significantly lower (*P* = 0.00024) in the hHSP27 group than in the controls (Figure 6A,B). Cytochrome c levels (15 kDa) in cytosolic fractions were also significantly lower (*P* = 0.00016) in the hHSP27 group vs. controls (Figure 6C,D). Because expansion of the damaged area following an ischemic insult has been attributed to immediate and direct cytotoxic effects of oxidative products [28], [29], we examined the effects of hHSP27 on levels of 8-hydroxydeoxyguanosine (8-OHdG), an oxidized nucleoside of DNA, and 4-hydroxy-2-hexenal (HHE), a major lipid peroxidation product. The numbers of cells immunopositive for these oxidative stress markers 24 h after reperfusion were significantly lower (*P*<0.001) in the hHSP27 group vs. controls (Figure 7A,B). The numbers of ionized calcium-binding adapter molecule-1 (Iba-1)-positive activated microglia and astrocytes 24 h after reperfusion, were also significantly lower (*P*<0.001) in the hHSP27 group vs. controls (Figure 7A,B).

## Discussion

In our experiments, delayed intravenous injections of phosphorylated, multimeric hHSP27 complexes following reperfusion after transient MCAO reduced infarct volume, neurological deficits, and apoptotic cell death, and at the same time decreased TUNEL reactions and the levels of cytochrome c, cleaved caspase-9, and cleaved caspase-3. The hHSP27 complex also decreased oxidative DNA damage, lipid peroxidation, and glial activation. Thus, hHSP27 appears to protect brain by inhibiting apoptosis and oxidative stress following ischemia and reperfusion. We also confirmed that it was the HSP27 that protected the brain, as a specific anti-HSP27 antibody inhibited the protective effects. Administered hHSP27 crossed the blood-brain barrier injured by ischemic insults and was localized in neurons on the ischemic side of brains, where it prevented apoptosis and oxidative stress and thereby neuronal death.

The more physiological hHSP27 (i.e., the phosphorylated multimeric complex) and not rHSP27 protected ischemic brains against damage. HSP27 tends to form variable-sized oligomers[30]–[32], depending on posttranslational modifications. Phosphorylation leads to the formation of small oligomers [30], [31], [33], monomers, and dimers [34], [35], which act as molecular chaperones, interfere with caspase activation, modulate oxidative stress, and regulate the cytoskeleton [7]. hHSP27 formed more dimers, tetramers, and multimers and less large oligomers than rHSP27. Dephosphorylation of hHSP27 increased the large oligomers and decreased the dimers, tetramers, and multimers, resulting in a loss of its protective effects. Phosphorylation may be a key factor in brain protection. Our results suggested that the injection of phosphorylated dimeric, tetrameric, and multimeric hHSP27 was important for brain protection in our model of ischemia/reperfusion. We speculated that intravenously injected hHSP27 may not only increase the concentration of endogenous HSP25, which is the mouse homolog of HSP27, but also may have a novel exposure effect to the neurons. hHSP27 was also localized in extracellular space in brain, and usually the concentration of HSP27 was very low in the extracellualr space. Further studies will be needed to ascertain the mechanisms by which intravenously injected hHSP27 provides neuronal cell protection.

Isolated hHSP27 also contained small amounts of αβ-crystalline and HSP20, which were part of high molecular weight HSP27 oligomers. Because they were co-purified with antibodies specific for HSP27 suggests that they were not simple contaminations, but that they interact with HSP27 in a sort of hHSP27 complex. Previous studies showed that because of similarity in structure and properties, HSP27, αβ-crystallin, and HSP20 are co-purified [36]; thus, the possibility exists that the αβ-crystallin and HSP20, which are part of the hHSP27 complex, may influence the effects of brain protection. We showed, however, that co-administration of hHSP27 in the presence of a specific anti-HSP27 antibody decreased the ability of hHSP27 to protect the brain against ischemic injury, strongly suggesting that most of the brain protective effect of hHSP27 was caused by HSP27. The necessary modifications of rHSP27, including phosphorylation and interaction with αβ-crystalline and HSP20, to mimic hHSP27 in ischemic brain treatment need to be identified.

The blood brain barrier (BBB) controls the passage of substances from the blood into the CNS [37]. Usually, injected proteins are hampered from reaching brain neurons by the tight regulation of the BBB. We observed peripherally injected FITC-HSP27 in neurons, indicating that FITC-HSP27 crossed the BBB and then entered neurons. Increased numbers of FITC-hHSP27-positive neurons on the ischemic side of the brain compared to the non-ischemic side suggests that the injured BBB enabled the FITC-hHSP27 to pass. Further studies are needed to elucidate the mechanism by which hHSP27 crosses the injured BBB and enters neurons.

HSP27 was shown to attenuate ischemic brain damage in transgenic mice overexpressing HSP27 [18], [38] and when it was delivered via viral HSP27 expression vectors [20], [39] or injected as the PEP-1-HSP27 protein [40]. There are, however, some differences between our study and the PEP-1-HSP27 study. An et al. used a delayed mouse model of neuronal cell death, a special ischemic model, whereby delayed neuronal death, caused by a very short 5-min artery occlusion, is mainly observed only in the hippocampus, and PEP-1-HSP27 was injected before the ischemic insult. By contrast, we used the more usual MCAO model of ischemia and administered hHSP27 after the ischemic insult, which is closer to the treatment paradigm that patients with ischemic stroke would experience. Ischemic damage was suppressed by the delayed, intravenous administration of hHSP27 after MCAO, as it would be administered to patients with brain infarctions. Although a delay of 1 h was more effective than 3 h, which would be difficult to accomplish in ischemic stroke patients, the necessary administration times may be different in human patients than in mice. Administering HSP27 viral vectors to patients may be dangerous, because HSP27 levels are significantly increased in many tumors [41], [42], [43], [44] and increased HSP27 expression correlates with increased resistance to cytotoxic (antineoplastic) compounds [41], [42], [45].

Because hHSP27 was purified from human tissues, the HSP27 effects in humans should not be affected by interspecies influences. Hence, hHSP27 has potential as a medical intervention to suppress cell death in ischemic stroke patients. We propose that delayed injection of human-derived HSP27 may salvage brain tissue and improve function following cerebral ischemia as well as other vascular diseases, such as cardiovascular disease. In the future, we intend to purify hHSP27 from a patient with acute stroke and subsequently inject the hHSP27 solution into the patient.
